# Host histone acetylation unlocks HDAC inhibitor potential

**DOI:** 10.18632/oncotarget.22422

**Published:** 2017-11-13

**Authors:** Manuela Terranova-Barberio, Scott Thomas, Pamela N. Munster

**Affiliations:** Pamela N. Munster: Department of Medicine, Division of Hematology and Oncology, Helen Diller Family Cancer Center, University of California, San Francisco, CA, USA

**Keywords:** HDAC inhibitors, epigenetic modulation, histone acetylation, HDAC2, PBMC

Histone deacetylase inhibitors (HDACi) are a complex class of anticancer therapeutics that have been in preclinical and clinical development for their ability to control gene expression through epigenetic modifications of histone and non-histone proteins. Although vorinostat, panobinostat and belinostat are approved as single agents in hematological tumors, HDACi efficacy as single agents in solid tumor malignancies has been modest. Much effort has, therefore, been placed in exploring the role of epigenetic modulation in combination with other therapeutic strategies to either prime existing therapy, such as immune therapy, or reverse acquired therapy resistance.

The broad effects of HDACi on protein acetylation cause extensive biological and cellular effects. Not surprisingly, this restricts the therapeutic window of HDACi. Hence, when integrating these agents into combinatorial strategies, careful attention should be directed towards the selection of target clients and overlapping toxicities. Furthermore, the approved single agent dose and schedule should be reconsidered. Recent examples of integrating HDACi with standard therapy to reverse resistance have been shown in breast cancer with exemestane [1] and tamoxifen [2], and in renal cell carcinoma with the VEGF/TKI inhibitor pazopanib [3]. These studies showed benefits in patients who have previously progressed on hormone therapy and VEGF directed therapy.

Emerging data from these studies pointed to the relevance of HDAC2 expression in the degree of histones acetylation and as a potential biomarker. In both breast cancer studies, accompanying correlative studies suggest that efficacy was considerably more pronounced in patients with an ability to acetylate H3 and H4 histone [1, 2]. Peripheral blood mononuclear cells (PBMCs) acetylation was found to be an acceptable surrogate for tumor acetylation. In many previous studies, histone acetylation has shown considerable inter-patient variability and has not consistently correlated with drug and plasma levels, across different HDACi and patient settings. Histone acetylation occurs briskly and then decreases sharply in many patients.

Moreover the ability to sustain histone H3 and H4 acetylation, beyond the pharmacological half-life of HDACi, was found to correlate with response, clinical benefit and time to progression [2-4]. Plasma levels, HDACi doses and concentrations were instead not robustly associated with histone acetylation or response [2, 4]. Furthermore, the inability to acetylate histones at all or maintain histone acetylation beyond the plasma HDACi half-lives in select patients, despite achieving high plasma concentrations, suggests that host-medicated factors influence histone acetylation.

The relevance of sustained acetylation was further shown in a recent phase I trial with considerable activity in heavily pretreated patients, where the HDACi abexinostat was combined with pazopanib. This study by Aggarwal *et al.*, showed durable benefits (beyond 2 years) in multiple patients, despite progression on prior VEGF inhibitors and other agents [3]. The tolerability of the combination of abexinostat and pazopanib for extended treatment is noteworthy and further speaks to the relevance of prolonged target modulation by the HDACi beyond its plasma half-life. In this trial, the exposure to abexinostat was reduced to only 4 doses per week, which considerably reduced on-target as well as off-target toxicities, and allowed continued dosing of the combination in patients, now exceeding 5 years [3]. A putative explanation to such prolonged response was the expression of high levels of the appropriate HDAC target in responders, allowing for an increase in pathway modulation with acceptable toxicities, whereas non-responders lacked sufficient HDAC expression. In particular, HDAC2 expression at baseline was identified as a predominant target for abexinostat and as a strong predictor of biological activity and clinical benefit [3]. Preclinical and clinical data support the role of HDAC2 as one of the most critical HDAC isoform for HDAC inhibition, being involved in VEGF [3] and ER [2] signaling and in DNA condensation [4, 5].

In most of the studies, the focus has been on HDAC2 as the major relevant pharmacological target, however it should be noted that several HDACs exhibit overlapping and compensatory activity. While HDAC6 baseline expression did not correlate with clinical outcomes in several studies, the expression of HDAC1 has not been extensively studied as a HDAC target and as a biomarker for response.

These studies highlighted two important and novel insights regarding the use of HDACi: i) the relevance of HDAC2 as a therapeutic target for HDACi and ii) the role of HDAC2 as a potential biomarker of response. Our studies further suggested that HDAC2 positive predictive value is correlated to its expression in PBMCs, hence HDAC2 expression appears to be predetermined by the host rather than the tumor, and likely epigenetically conserved in patients.

These findings are further supported by data showing that tumor expression of HDAC1 and HDAC2 was found to be associated with worse overall survival in both gastric and breast cancer [6, 7]. However, there are no conclusive studies evaluating HDAC2 tumor expression as a response predictor. Little is known about the evolution of HDAC2 expression in tumors compared to adjacent normal tissue or PBMCs, and none of the HDACi trials clearly demonstrated changes of HDAC expression in response to therapy.

Given the strong correlation between HDAC2 expression, sustained acetylation and response to HDACi, future work should focus on developing new technologies to identify patients with an intrinsic ability for hyper acetylation when treated with epigenetic modifiers in real time and ideally prior to enrollment [8]. Such methodologies will have a great impact in future studies, especially as epigenetic modifiers will enter combination strategies with immune-oncology and the initially proposed doses and schedule may not be appropriate when studying the effects on immune cells.
